# Partial hepatectomy for a patient with Rendu–Osler–Weber disease: a case report

**DOI:** 10.1186/s40792-023-01588-w

**Published:** 2023-01-19

**Authors:** Naoko Sekiguchi, Daisaku Yamada, Shogo Kobayashi, Kazuki Sasaki, Yoshifumi Iwagami, Yoshito Tomimaru, Takehiro Noda, Hidenori Takahashi, Yuichiro Doki, Hidetoshi Eguchi

**Affiliations:** Department of Gastroenterological Surgery, Graduate School of Medicine, Osaka University, 2-2-E2, Yamadaoka, Suita, Osaka 565-0871 Japan

**Keywords:** Osler disease, Hepatectomy, Delayed postoperative hemorrhage (DPH)

## Abstract

**Background:**

Rendu–Osler–Weber disease (Osler disease) is a genetic disease with an autosomal dominant inheritance pattern. It is characterized by widespread telangiectasia in multiple organs. Liver involvement of FNH is relatively common, but liver cancer is very rare, and there are few reports on hepatectomy or postoperative complications. We report a very rare case in which hepatectomy was performed for a patient with Osler disease.

**Case presentation:**

The patient was a 39-year-old man with Osler disease who had been previously diagnosed with multiple FNH and who had been followed for 8 years. During follow-up, the diameter of an S6 lesion gradually increased from 30 to 50 mm; no other lesions increased in size. We decided to perform partial liver resection as total biopsy for the growing tumor, due to the possibility that the growing tumor lesion included malignant components. The pathological examination revealed no obvious malignancy, which was finally diagnosed FNH. The postoperative course was uneventful and he was discharged on the 14th day after surgery. In the second month after discharge, he was transferred to our hospital with sudden abdominal pain in the right hypochondrium with severe tenderness. CT showed extravasation of contrast medium from the hepatic dissection surface in S6, and the hematoma extended to the pelvic floor. Emergency IVR was performed and revealed leakage of the contrast medium from the A6 branch. We embolized the A6 with Lipiodol. After embolization, there were no major problems, and the patient was discharged on the 9th day after the treatment.

**Conclusions:**

Postoperative hemorrhage often occurs within 24 h after surgery, and 2 months after surgery is considered to be the late stage of the wound healing process, and postoperative hemorrhage at this timepoint is considered rare. This unexpected delayed postoperative hemorrhage may have been related to the etiology and pathology of Osler disease, nevertheless, case reports of hepatectomy for patients with Osler disease are limited. We, therefore, report the present case with a review of the relevant literature.

## Background

Rendu–Osler–Weber disease (Osler disease) is a genetic disease with an autosomal dominant inheritance pattern, characterized by widespread telangiectasis that can involve the skin, mucous membranes, lung, brain, gastrointestinal tract and/or liver [1]. It has an estimated prevalence of 1–2 cases per 10,000 in the Japanese population [2]. In patients with this condition, the post-capillary venules are dilated and fuse with arterioles, bypassing the capillary network, resulting in arterio-venous communication and telangiectasia [3]. Telangiectasia with Osler disease is a rare systemic fibrovascular dysplasia that bears—as basic defect—an alteration in the elastic and muscle layers of the vessel walls, making them more vulnerable to spontaneous rupture and injury [4]. Vascular histopathological features include dilation of capillaries, dilation of venules, underdevelopment of the muscle layer of arterioles, and thinning of venule endothelial cells. In addition, there is a defect in the internal elastic lamina and smooth muscle. It is also possible for aneurysms to form in the wall of the regenerated artery and rupture. Thus, the dominant symptom of Osler disease is hemorrhage from telangiectasia; meanwhile, focal nodular hyperplasia (FNH) represents the major liver involvement of Osler disease, occurring in 2.9% of cases. Reports on liver cancer in patients with Osler disease are very rare, and there are few reports on hepatectomy or postoperative complications.

We herein report the case of a patient with Osler disease who underwent partial hepatectomy for a lesion that was suspected to be hepatocellular carcinoma (HCC), in whom postoperative hemorrhage occurred 2 months after surgery.

## Case presentation

**Fig. 1:**
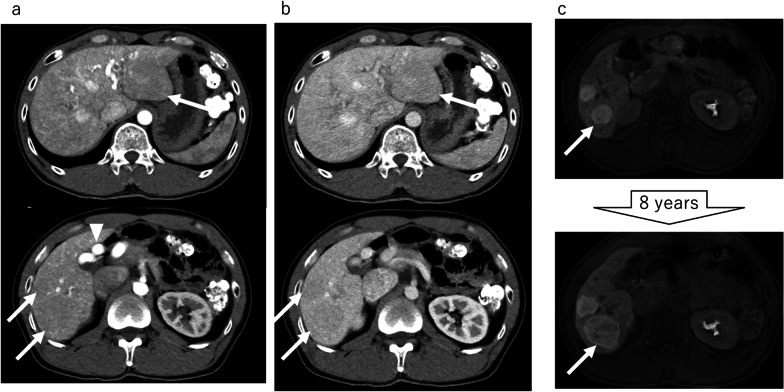
Preoperative images. At the initial visit, CT showed multiple lesions of up to 75 mm in both lobes of the liver (arrows). In the arterial phase, the common and proper hepatic artery (arrowhead) was markedly dilated and tortuous. The lesions were slightly enhanced, and the liver parenchyma was strongly enhanced from the arterial phase and was very heterogeneous due to small telangiectasias (**a**). In the portal phase, the lesions showed delayed enhancement (**b**). MRI showed that lesions were enhanced in the hepatocellular phase. During 8 years of follow-up, the only lesion to increase in size was a segment 6 lesion (arrow); this tumor increased in diameter from 30 to 50 mm (**c**)

The patient was a 39-year-old man with Osler disease, who had previously been diagnosed with multiple FNH and who had been followed for 8 years. Contrast-enhanced computed tomography (CT) at the initial visit revealed that the common and proper hepatic artery was markedly dilated and tortuous, and the liver parenchyma was strongly enhanced from the arterial phase and was very heterogeneous due to small telangiectasias (Fig. 1a). Twelve masses of up to 78 mm in diameter were located in both lobes of the liver, and each mass showed mild enhancement with a delayed contrast effect (Fig. 1b). Gadolinium-ethoxybenzyl-diethylenetriamine penta-acetic acid enhanced magnetic resonance imaging (Gd-EOB–MRI) showed similar findings to CT, with all lesions enhanced in the hepatocellular phase. On imaging obtained during 8 years of follow-up, a segment 6 (S6) lesion was the only lesion to increase in size, with the tumor diameter gradually increasing from 30 to 50 mm (Fig. 1c, upper figure:initial visit, lower figure: after 8 years of follow-up). On CT, the tumor growth was unclear due to extensive shunt formation, and size changes could not be confirmed. The shunt proliferation was almost no change. Percutaneous liver puncture was not performed to decide the indications for hepatectomy, because a negative biopsy result would not prove the absence of malignancy in the tumor mass, besides, there the procedure was associated with a certain risk of hemorrhage due to Osler disease. We, therefore, decided to perform liver resection as total biopsy for the growing tumor, due to the possibility that malignant components were present in the growing tumor lesion. Due to the development of intrahepatic arteriovenous shunt, we opted to perform partial resection of S6 by laparotomy to secure the patient’s safety.

When the patient was hospitalized for surgery, he had not taken any medications or nutritional supplements. He had no relevant past medical history or family history, and tests for hepatitis B and C were negative. On physical examination, there were no remarkable findings. Laboratory tests on admission showed a few abnormal results. As follows: γ-glutamyl transpeptidase, 200 U/L; alkaline phosphatase,132 U/L; and indocyanine green retention rate at 15 min (ICG-R15), 30%. Since it was assumed that the increase of ICG-R15 may have been influenced by the presence of arteriovenous shunts in liver, asialoglycoprotein receptor imaging was performed. Radio isotope (RI) accumulation in the liver was reduced, at 0.865 (normal range 0.924–0.959), indicating mild liver damage. Tumor markers were normal at all preoperative stage. Patients with Osler disease often show shunt formation in other organs, but in this case, there were no obvious arteriovenous shunts on echocardiography, brain MRI, and pulmonary–vascular scintigraphy. Fluorodeoxyglucose–positron emission tomography (FDG–PET) showed no abnormal uptake in the liver or other organs.

**Fig. 2:**
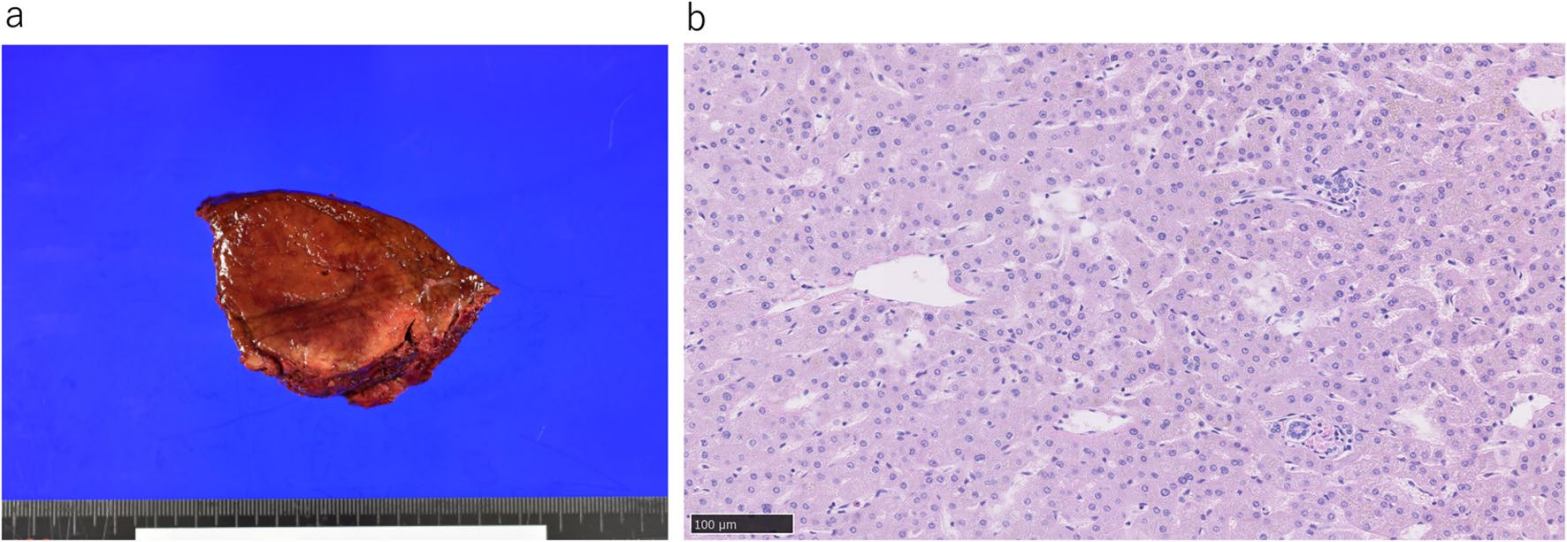
Resected specimen and pathological findings. Observation of the resected specimen revealed that the tumor was yellow to brown with a well-defined border (**a**). In the histopathological examination, the lesion was 70 mm in diameter. There were no malignant findings in hepatocytes. In general, the portal region consists of three elements: the artery, portal vein and bile duct. However, in the portal region of this case, where the tumor was located, these components were absent and scattered small blood vessels were observed. Scale bar 100 µm (**b**)

In surgical findings, we recognized many telangiectasia on the surface of liver. We performed liver resection using the Pringle maneuver procedure; however, bleeding from the hepatic vein was conspicuous during surgery, possibly because the venous pressure remained high due to telangiectasia. For veins of ≤ 7 mm during hepatectomy, a vessel sealing device was used. Surgery proceeded without any major problems; the operative time was 147 min and the total bleeding volume was 220 ml. Observation of the resected specimen showed that the tumor was yellow to brown with a well-defined border (Fig. 2a). A pathological examination revealed no obvious malignancy. The final diagnosis was FNH. In general, the portal region consists of three elements: the artery, portal vein and bile duct. However, in the portal region of this case, where the tumor was located, these components were absent and scattered small blood vessels were observed (Fig. 2b).

There were no complications in the time from surgery until the first discharge from our hospital. In blood tests at 7 days after surgery, the CRP level had returned to normal but the D-dimer level remained mild high. Contrast-enhanced CT was performed for the purpose of evaluating hepatic blood flow and excluding venous thrombosis. We confirmed that the hepatic blood flow was normal and that there was no venous thrombosis. Hence, we considered that the increase in D-dimer was a postoperative change. The patient was discharged 14 days after surgery. At an outpatient visit 7 days after discharge, the D-dimer level had returned to normal and there was no increase in the inflammatory response.

**Fig. 3:**
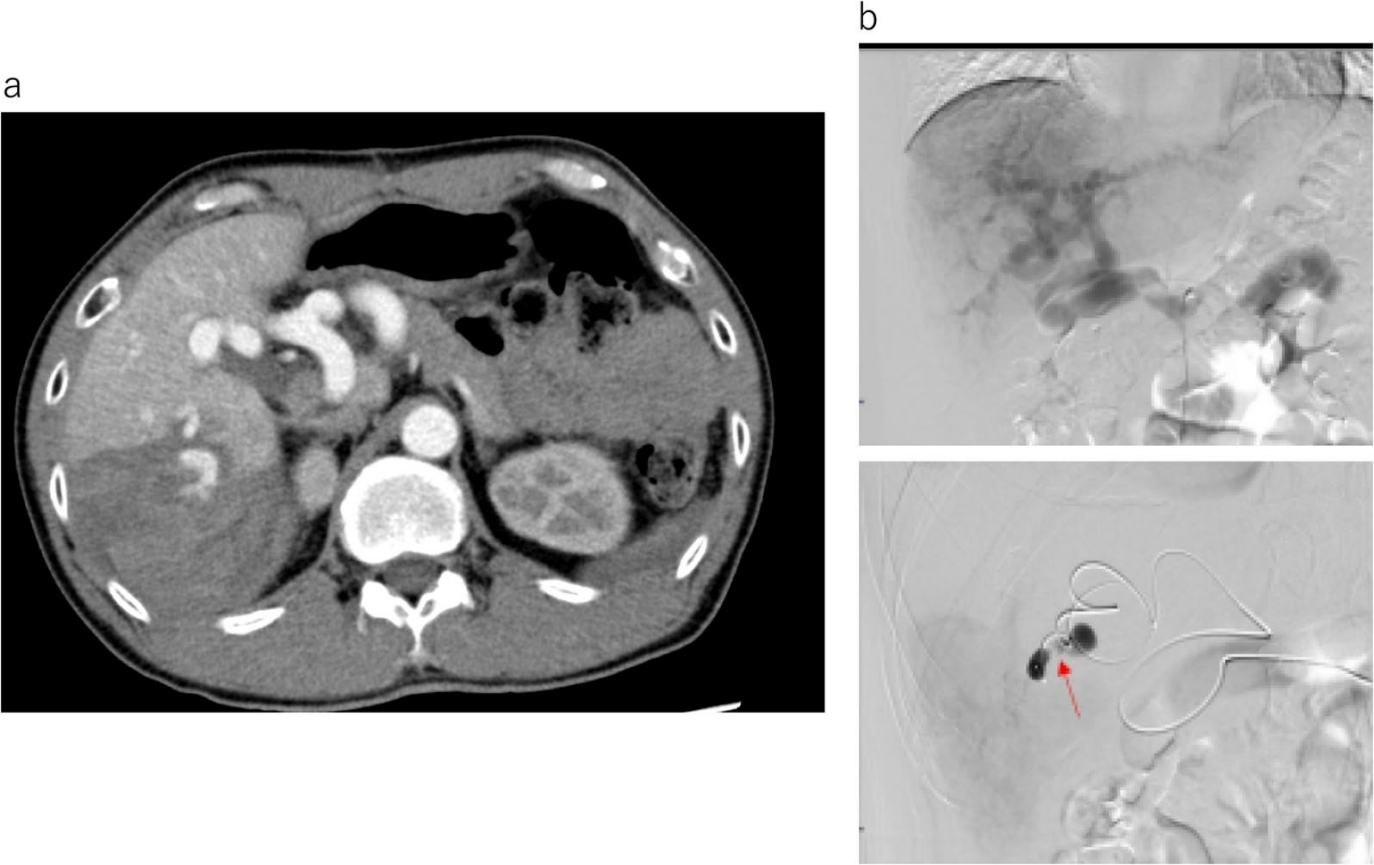
CT and interventional radiology (IVR) at abdominal hemorrhage. Enhanced-computed tomography (CT) shows poor liver regeneration of the right lobe, exposed blood vessels on the resected surface of the liver, and contrast medium leaking from the blood vessels on the resected surface of the liver into the abdominal cavity (**a**). An angiographic image approaching from the right external iliac artery (**b**). The bleeding site could not be identified by contrast imaging from the celiac artery root, because the hepatic artery was dilated and the flow velocity was fast (upper panel). When A6 was selected, the site of contrast medium leakage was confirmed (lower panel)

At 2 months after discharge, the patient was transferred to our hospital due to sudden abdominal pain in the right hypochondrium with severe tenderness. On physical examination, his blood pressure was 143/72 mmHg, his pulse was 73 beats/min, and he showed diaphoresis. His laboratory test data showed no marked changes from his last blood test data (Table 1). Since he had previously undergone laparotomy, contrast-enhanced CT was performed to investigate adhesive ileus or strangulation ileus. CT showed the extravasation of contrast medium from the hepatic S6 dissection surface, and the hematoma extended to the pelvic floor (Fig. 3a). During CT, the patient developed tachycardia and shock, and emergency interventional radiology (IVR) was immediately performed. IVR revealed the leakage of the contrast medium from A6 branch was observed (Fig. 3b). Vascular embolization was performed using Lipiodol from the root of A6, and embolization by Lipiodol at the bleeding site was confirmed by CT. After embolization, there were no major problems, and the patient was discharged on the 9th day after treatment. The patient spends common day life for 1 year after the discharge without major complications.

**Table 1:** Laboratory test at admission, first visit after discharge, and emergency transportation

	Initial visit	1 month after surgery	Emergency transportation	
WBC	5140	4210	5470	/µL
RBC	505	442	559	10^4^/µL
Hb	16.3	14.4	14.9	g/dL
Ht	47.2	42	42.5	%
PLT	21.1	22.2	20.3	10^4^/µL
PT	76	75	75	%
APTT	34	32	33	seconds
Na	142	144	141	mmol/L
K	3.7	3.9	3.6	mmol/L
Cl	103	106	105	mmol/L
Alb	4.7	4.3	4.2	g/dL
UN	14	13	13	mg/dL
Cre	0.75	0.65	0.67	mg/dL
eGFR	92.9	101.7	105.1	mL/min
AST	38	25	47	U/L
ALT	37	20	34	U/L
γGTP	200	116	117	U/L
ALP	132	157	148	U/L
T-Bil	2.2	1.4	1.3	mg/dL
D-Bil	0.5	0.4	0.4	mg/dL
I-Bil	1.7	1	0.9	mg/dL
CRP	< 0.04	0.06	0.06	mg/dL

Laboratory tests showed mild elevation of hepatobiliary enzymes. The CRP level was normal at all times

## Discussion

Osler disease is caused by mutations in one of two genes. Accordingly, it is subclassification into type 1 and 2. Both genes encode transmembrane proteins involved in the transforming growth factor (TGF)-β signaling pathway and are expressed predominantly on the vascular endothelium [5]. In type 1, the mutation is located on chromosome 9 in the gene that encodes endoglin (ENG), a type III TGF-β receptor [6]. In type 2, the mutation is located on chromosome 12, in the gene that encodes activin receptor-like kinase type I (ALK-1 or ACVRL1), a type I TGF-β receptor [7]. Although the genotypic–phenotypic correlations are not yet fully defined, it appears that liver involvement is more prevalent in type 2 [8]. The patient in this report had a history of recurrent epistaxis and dilatation of the peripheral blood vessels on his skin. Hence, he was diagnosed with Osler disease based on the Curacao criteria; however, the type was unknown, because the patient did not receive a genetic test.

Liver involvement is observed in 67–84% of Osler disease patients [9]. The pathophysiology, which mediates the development liver involvement, is due to an imbalance between hepatic artery and portal venous blood flow and ultimately leads to increased hepatic arterial inflow [10–12]. The flow imbalance in the liver parenchyma facilitates the development of hepatocellular over-regenerative activity, and nodular regenerative hyperplasia (NRH) or focal nodular hyperplasia (FNH) often develop in the liver of patients with Osler disease [13, 14]. Among these patients, the prevalence of FNH is 2.9% (100-fold greater than in the general population) [15]. Although FNH is a benign liver lesion, it is sometimes difficult to distinguish from hepatocellular carcinoma (HCC), and its presence can lead to diagnostic confusion in Osler disease patients, because typical findings of HCC on dynamic CT scan can be masked by widespread arteria–venous shunts in the liver [16].

There is no established opinion regarding the development of HCC in Osler disease patients. On one hand, it is assumed that the imbalance between the hepatic artery and portal vein induces an increase in the level of hepatic growth factors, and the sequential reactions may facilitate the development of HCC [11, 12]. On the other hand, it was reported that Osler disease patients with no other risk factors rarely develop HCC [17], or develop HCC less frequently in comparison with the general population [18]. A search of the relevant literature using the PubMed database (key words: Osler disease and hepatocellular carcinoma) yielded only five reports on HCC in Osler disease, and 3 of these 5 cases had other risk factors for the development of HCC (e.g., hepatitis) [19–23].

For liver lesions, percutaneous liver biopsy is usually selected when the tumor size increases and malignancy cannot be ruled out. Although a past case series reported that 6 Osler disease patients underwent percutaneous biopsy without any complications [24], percutaneous liver biopsy is generally avoided for the diagnosis and treatment as the procedure is associated with a high risk of bleeding. In our case, malignancy was suspected, because the size increased during 8 years, and total biopsy was performed surgically. In the past reports, only three cases underwent hepatectomy and postoperative complications occurred in two cases. One patient had ascites, and the other had hepatic vein thrombosis. In both cases, the complications improved with medication [22, 25]. In the present case, we determined our approach after giving the patient a detailed explanation of the surgical risks. As a result, the perioperative period was uneventful and the pathological findings of the resected specimen showed no malignancy and the diagnosis was FNH.

Postoperative hemorrhage often occurs within 24 h after surgery. Two months after surgery is considered to be the late stage of the wound healing process, and postoperative hemorrhage at this timepoint is considered rare. Delayed postoperative hemorrhage (DPH) is defined as hemorrhage observed more the 24 h postoperatively. A past report suggested that the incidence rate of DPH in hepatobiliary and pancreatic surgery was 1.5% [26]. Most cases occurred in association with infection. In our case, the patient did not show an increased inflammatory response, there was no accumulation of fluid on the resected liver surface, and there were no complications, such as bile leakage. However, at 2 months after the surgery, hemorrhage from an artery on the resected surface of the liver occurred. Therefore, we assumed that the etiology of DPH might be related to Osler disease.

CT at the time of bleeding showed poor liver regeneration, and the site of vascular resection was exposed on the surface of the liver. Telangiectasia were scattered in whole liver same as preoperatively, and we could not reveal that there was shunt formation in the bleeding site. The comparison of CT scans before and after postoperative bleeding revealed that the periphery of the right hepatic vein ran around the area, where the extravasation of the contrast medium is observed. Thus, we hypothesized that the mechanism of DPH was as follows. First, the vein running on the surface of the liver fused with a hepatic peripheral artery belonging to the A6 branch. Subsequently, shunt formation gradually increased the venous pressure, and sealing of the vein by the sealing device might not have been tolerated. Besides, the blood vessels of patients with this disease are in extremely fragile. Eventually, the vein on the liver surface—which also showed hepatic artery shunt—ruptured. Additional suturing was considered during surgery when hemostasis was difficult under common surgical procedure of hepatectomy, nevertheless, it was not necessary and we felt relieved at the time of finish surgery. However, we experienced this case in which DPH occurred, and we regret that we might have to ligate all vessels.

Although the exact cause of this episode was unknown, this case represents a valuable example that demonstrates the risk of hemorrhage in patients with Osler disease after hepatectomy, even if there are no complications during the perioperative period.

## Conclusions

We experienced a case of partial hepatectomy for Osler disease. The patient showed with delayed intra-abdominal hemorrhage at 2 months after surgery. The long-term risk of bleeding should be considered when managing patients with vascular dysplasia, such as Osler disease.

## Data Availability

The data that support the findings of this study are available from the corresponding author upon reasonable request.

## Abbreviations

FNH: Focal nodular hyperplasia
HCC: Hepatocellular carcinoma
CT: Computed tomography
Gd-EOB–MRI: Gadolinium-ethoxybenzyl-diethylenetriamine penta-acetic acid enhanced magnetic resonance imaging
ICG-R15: Indocyanine green retention rate at 15 min
RI: Radio isotope
FDG–PET: Fluorodeoxyglucose–positron emission tomography
CRP: C-reactive protein
IVR: Interventional radiology
TGF-β: Transforming growth factor β
ENG: Endoglin
ALK-1/ACVRL1: Activin receptor-like kinase type I
NRH: Nodular regenerative hyperplasia
DPH: Delayed postoperative hemorrhage
